# Supplementary material for: Low Mediterranean Diet Adherence Is Associated with Poor Socioeconomic Status and Quality of Life: A Cross-Sectional Analysis
Source: Nutrients. 2025 Mar 5;17(5):906. doi: 10.3390/nu17050906 (PMC11901638; doi:10.3390/nu17050906)
Supplement: Supplementary file 1 [file nutrients-17-00906-s001.zip › nutrients-3439022-supplementary.pdf]

SUPPLEMENTAL MATERIALS

Supplementary S1: SF-36 questionnaire

THE MOS 36-ITEM SHORT-FORM HEALTH SURVEY (SF-36)

INSTRUCTIONS: This survey asks for your views about your health. This information will help keep track of how you feel and how well you are able to do your usual activities.

Answer every question by marking the answer as indicated. If you are unsure about how to answer a question, please give the best answer you can.

1. In general, would you say your health is:

(circle one)

Excellent 1

Very good 2

Good 3

Fair 4

Poor 5

2. Compared to one year ago, how would you rate your health in general now?

(circle one)

Much better now than one year ago 1

Somewhat better now than one year ago 2

About the same as one year ago 3

Somewhat worse now than one year ago 4

Much worse now than one year ago 5

Copyright © 1992 MOS Trust, Inc.
All rights reserved.

3. The following items are about activities you might do during a typical day. Does your health now limit you in these activities? If so, how much?

(circle one number on each line)

**ACTIVITIES**	**Yes, Limited A Lot**	**Yes, Limited A Little**	**No, Not Limited At All**
a. **Vigorous activities**, such as running, lifting heavy objects, participating in strenuous sports	1	2	3
b. **Moderate activities**, such as moving a table, pushing a vacuum cleaner, bowling, or playing golf	1	2	3
c. Lifting or carrying groceries	1	2	3
d. Climbing **several** flights of stairs	1	2	3
e. Climbing **one** flight of stairs	1	2	3
f. Bending, kneeling, or stooping	1	2	3
g. Walking **more than a mile**	1	2	3
h. Walking **several blocks**	1	2	3
i. Walking **one block**	1	2	3
j. Bathing or dressing yourself	1	2	3

4. During the past 4 weeks, have you had any of the following problems with your work or other regular daily activities as a result of your physical health?

(circle one number on each line)

	**YES**	**NO**
a. Cut down on the **amount of time** you spent on work or other activities	1	2
b. **Accomplished less** than you would like	1	2
c. Were limited in the **kind** of work or other activities	1	2
d. Had **difficulty** performing the work or other activities (for example, it took extra effort)	1	2

Copyright © 1992 MOS Trust, Inc.
All rights reserved.

5. During the past 4 weeks, have you had any of the following problems with your work or other regular daily activities as a result of any emotional problems (such as feeling depressed or anxious)?

(circle one number on each line)

	YES	NO
a. Cut down the **amount of time** you spent on work or other activities	1	2
b. **Accomplished less** than you would like	1	2
c. Didn't do work or other activities as **carefully** as usual	1	2

6. During the past 4 weeks, to what extent has your physical health or emotional problems interfered with your normal social activities with family, friends, neighbors, or groups?

(circle one)

Not at all 1

Slightly 2

Moderately 3

Quite a bit 4

Extremely 5

7. How much bodily pain have you had during the past 4 weeks?

(circle one)

None 1

Very mild 2

Mild 3

Moderate 4

Severe 5

Very severe 6

Copyright © 1992 MOS Trust, Inc.
All rights reserved.

8. During the past 4 weeks, how much did pain interfere with your normal work (including both work outside the home and housework)?

(circle one)

Not at all 1

A little bit 2

Moderately 3

Quite a bit 4

Extremely 5

9. These questions are about how you feel and how things have been with you during the past 4 weeks. For each question, please give the one answer that comes closest to the way you have been feeling. How much of the time during the past 4 weeks -

(circle one number on each line)

	All of the Time	Most of the Time	A Good Bit of the Time	Some of the Time	A Little of the Time	None of the Time
a. Did you feel full of pep?	1	2	3	4	5	6
b. Have you been a very nervous person?	1	2	3	4	5	6
c. Have you felt so down in the dumps that nothing could cheer you up?	1	2	3	4	5	6
d. Have you felt calm and peaceful?	1	2	3	4	5	6
e. Did you have a lot of energy?	1	2	3	4	5	6
f. Have you felt downhearted and blue?	1	2	3	4	5	6
g. Did you feel worn out?	1	2	3	4	5	6
h. Have you been a happy person?	1	2	3	4	5	6
i. Did you feel tired?	1	2	3	4	5	6

Copyright © 1992 MOS Trust, Inc.
All rights reserved.

Supplementary S2: Scoring method—RAND 36-Item Health Survey 1.0.

Table S1

Step 1: Recoding Items

Item numbers	Change original response category *	To recoded value of:
1, 2, 20, 22, 34, 36	1 →	100
	2 →	75
	3 →	50
	4 →	25
	5 →	0
3, 4, 5, 6, 7, 8, 9, 10, 11, 12	1 →	0
	2 →	50
	3 →	100
13, 14, 15, 16, 17, 18, 19	1 →	0
	2 →	100
21, 23, 26, 27, 30	1 →	100
	2 →	80
	3 →	60
	4 →	40
	5 →	20
	6 →	0
24, 25, 28, 29, 31	1 →	0
	2 →	20
	3 →	40
	4 →	60
	5 →	80
	6 →	100
32, 33, 35	1 →	0
	2 →	25
	3 →	50
	4 →	75
	5 →	100

Table S2

Step 2: Averaging Items to Form Scales

Scale	Number of items	After recoding per Table 1, average the following items
Physical functioning	10	3 4 5 6 7 8 9 10 11 12
Role limitations due to physical health	4	13 14 15 16
Role limitations due to emotional problems	3	17 18 19
Energy/fatigue	4	23 27 29 31
Emotional well-being	5	24 25 26 28 30
Social functioning	2	20 32
Pain	2	21 22
General health	5	1 33 34 35 36

Table S3

Reliability, Central Tendency, and Variability of Scales in the Medical Outcomes Study

Scale	Items	Alpha	Mean	SD
Physical functioning	10	0.93	70.61	27.42
Role functioning/physical	4	0.84	52.97	40.78
Role functioning/emotional	3	0.83	65.78	40.71
Energy/fatigue	4	0.86	52.15	22.39
Emotional well-being	5	0.90	70.38	21.97
Social functioning	2	0.85	78.77	25.43
Pain	2	0.78	70.77	25.46
General health	5	0.78	56.99	21.11
Health change	1	—	59.14	23.12
